# Supplementary material for: Homeostats: The hidden rulers of ion homeostasis in plants
Source: Quant Plant Biol. 2024 Sep 3;5:e8. doi: 10.1017/qpb.2024.8 (PMC11706688; doi:10.1017/qpb.2024.8)
Supplement: Dreyer et al. supplementary material [file S2632882824000080sup001.pdf]

Supplementary Figure S1

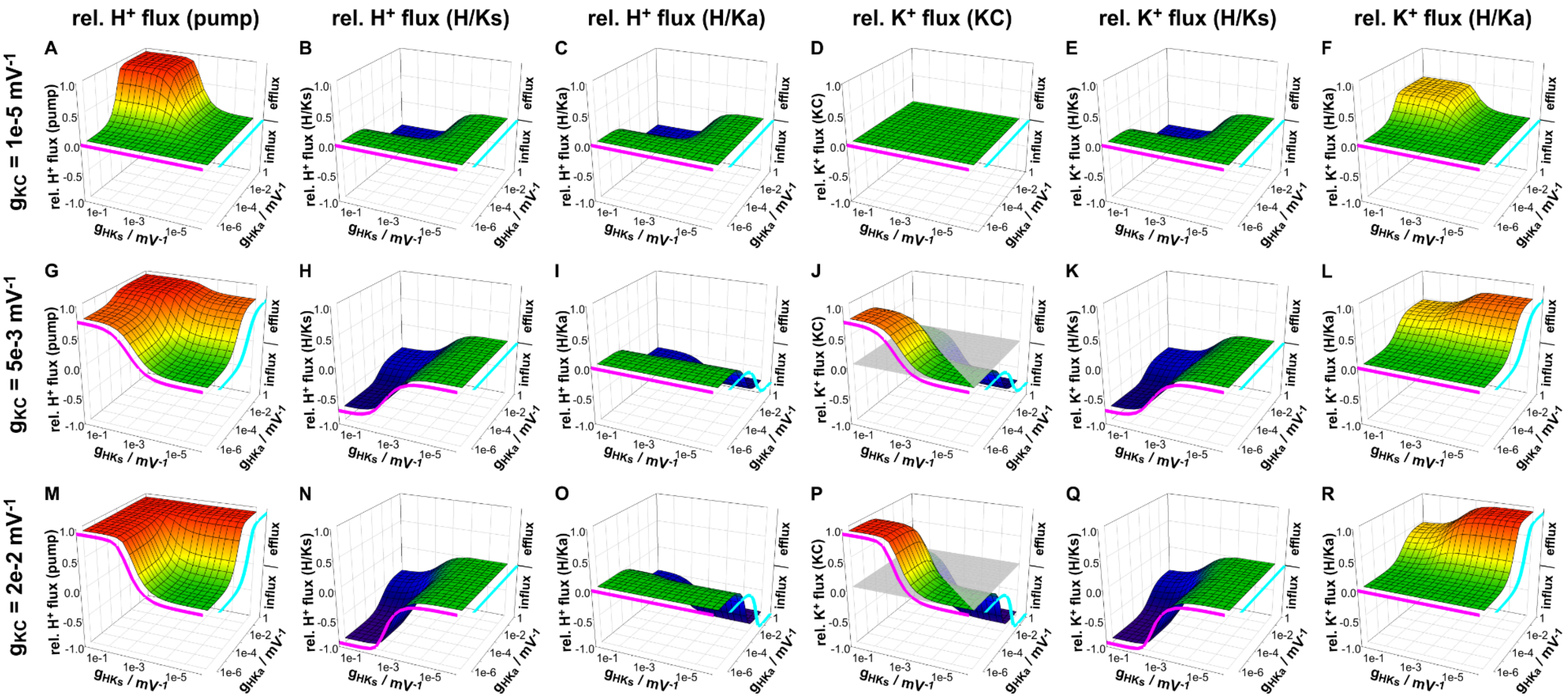

Figure S1. H^+ and K^+ fluxes through the different transporters in homeostatic (steady state) conditions. Dependency of the H^+ and K^+ fluxes on the activities of the K^+ channels (g_{KC}), H^+/K^+ symporters (g_{HKs}) and H^+/K^+ antiporters (g_{HKa}). (**A, G, M**) Relative H^+ flux mediated by the H^+ ATPase. (**B, H, N**) Relative H^+ flux mediated by the H^+/K^+ symporter. (**C, I, O**) Relative H^+ flux mediated by the H^+/K^+ antiporter. (**D, J, P**) Relative K^+ flux mediated by the K^+ channel. (**E, K, Q**) Relative K^+ flux mediated by the H^+/K^+ symporter. (**F, L, R**) Relative K^+ flux mediated by the H^+/K^+ antiporter. The fluxes are shown relative to the maximal H^+ efflux that can be generated by the H^+ ATPase ($J_{Hmax} = I_{Hmax}/e_0$). Data were calculated for the case $n_s = 1$, $n_a = 1$, $V_{0,pump} = -200$ mV, and $E_H = +57.6$ mV (ΔpH = 1). The magenta lines show the values in the absence of active H^+/K^+ antiporters ($g_{HKa} = 0$), whereas the cyan lines indicate the values in the absence of active H^+/K^+ symporters ($g_{HKs} = 0$).

Supplementary Figure S2

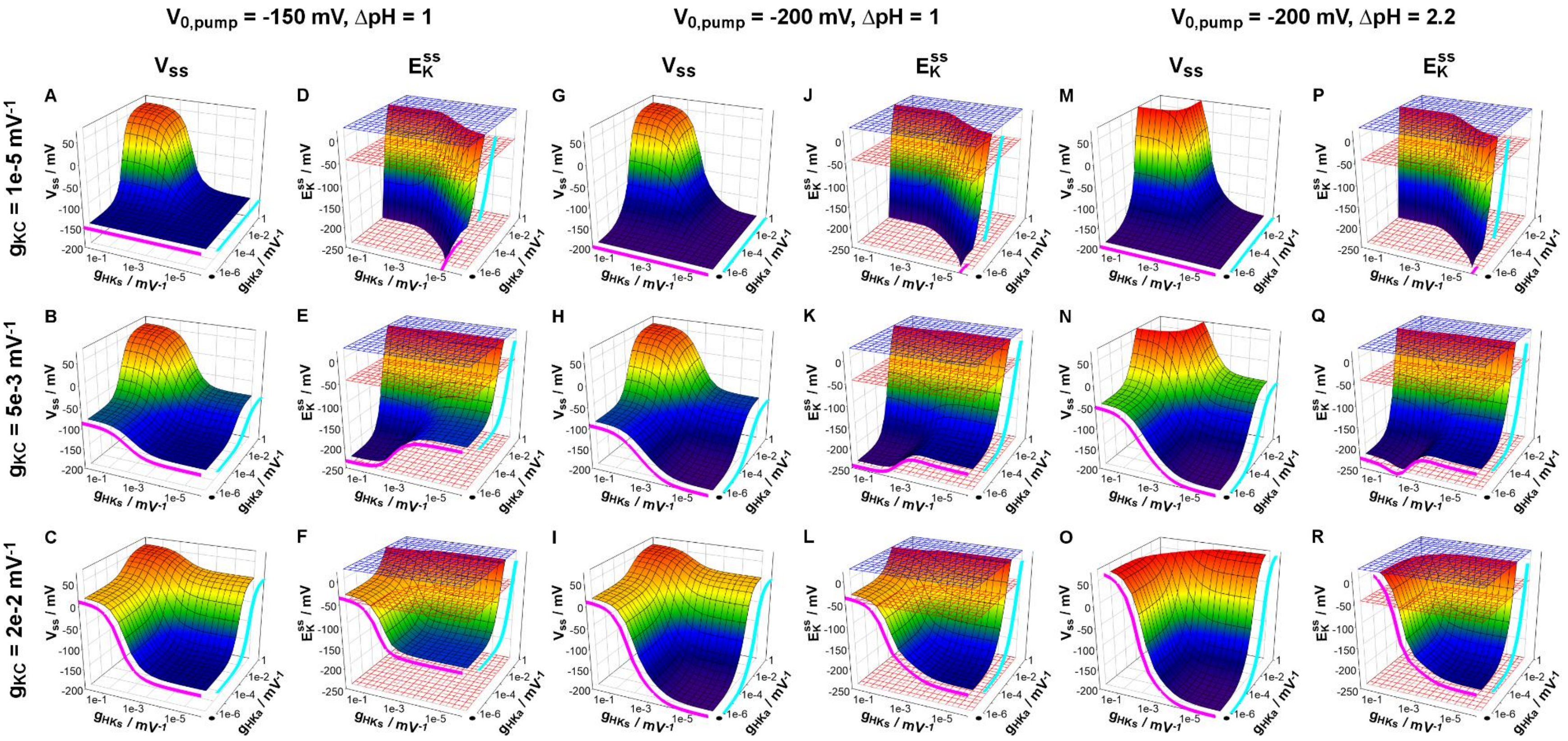

Figure S2. Membrane voltage and K⁺ gradient in homeostatic (steady state) conditions for different values of $V_{0,pump}$ and E_H. Membrane voltage (V_{ss}, **A-C**, **G-I**, **M-O**) and K⁺ gradient (E_K^{ss}, **D-F**, **J-L**, **P-R**) in steady state for selected values of the activity of K⁺ channels (g_{KC}), H⁺/K⁺ symporters (g_{HKs}) and H⁺/K⁺ antiporters (g_{HKa}). Data were calculated for the case n_s = 1, n_a = 1, with (**A-F**) $V_{0,pump}$ = -150 mV, and E_H = +57.6 mV (ΔpH = 1), (**G-L**) $V_{0,pump}$ = -200 mV, and E_H = +57.6 mV (ΔpH = 1), and (**M-R**) $V_{0,pump}$ = -200 mV, and E_H = +126.6 mV (ΔpH = 2.2). The magenta lines show the values in the absence of active H⁺/K⁺ antiporters (g_{HKa} = 0), whereas the cyan lines indicate the values in the absence of active H⁺/K⁺ symporters (g_{HKs} = 0). The blue and red meshes limit roughly the physiologically relevant ranges for E_K^{ss} (between +20 mV, i.e. $[K^+]_{out}/[K^+]_{in}$ ≈ 267mM/120mM and -50 mM, i.e. $[K^+]_{out}/[K^+]_{in}$ ≈ 16mM/120mM, for an endomembrane, and between -50 mV and -250 mV, i.e. $[K^+]_{out}/[K^+]_{in}$ ≈ 5µM/120mM, for the plasma membrane).

Supplementary Figure S3

$V_{0,pump}$ = -150 mV

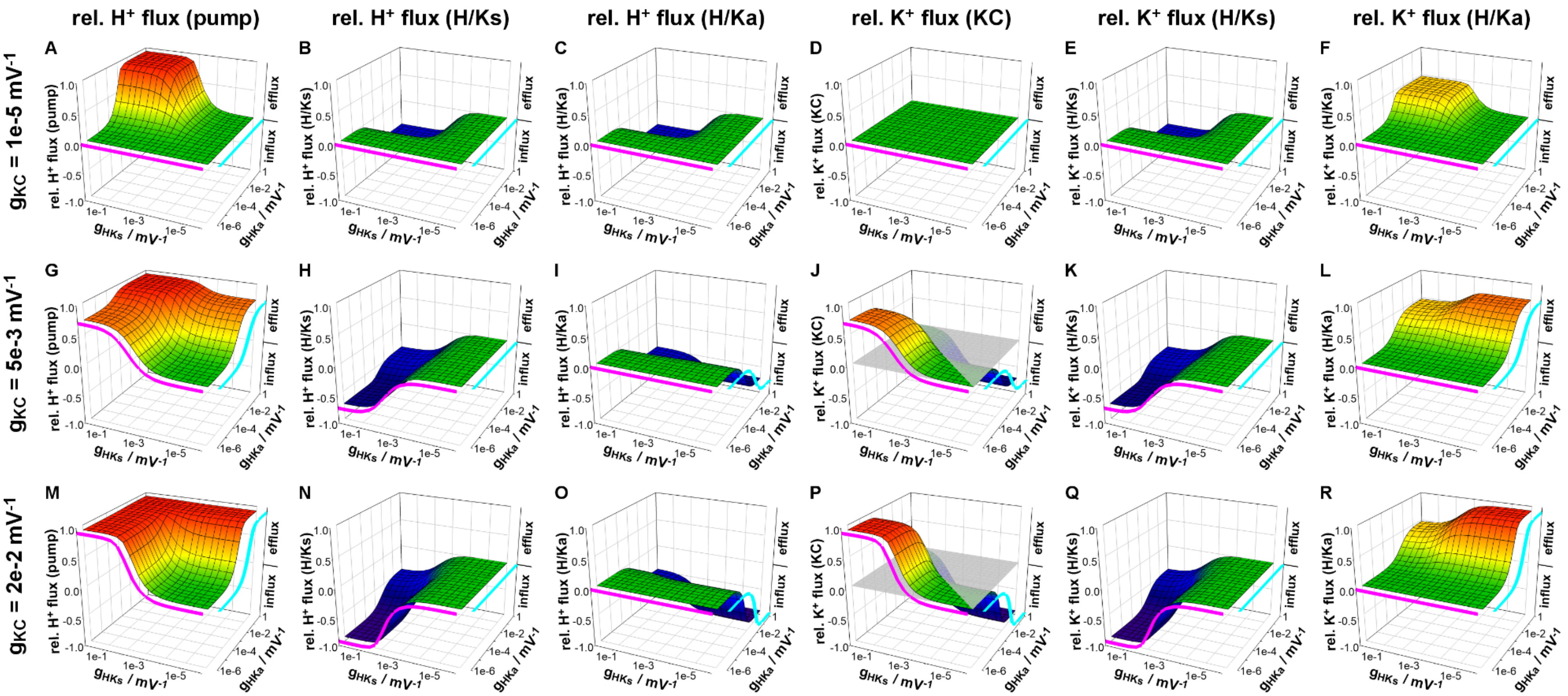

Figure S3. H^+ and K^+ fluxes through the different transporters in homeostatic (steady state) conditions for $V_{0,pump}$ = -150 mV. Dependency of the H^+ and K^+ fluxes on the activities of the K^+ channels (g_{KC}), H^+/K^+ symporters (g_{HKs}) and H^+/K^+ antiporters (g_{HKa}). (**A, G, M**) Relative H^+ flux mediated by the H^+ ATPase. (**B, H, N**) Relative H^+ flux mediated by the H^+/K^+ symporter. (**C, I, O**) Relative H^+ flux mediated by the H^+/K^+ antiporter. (**D, J, P**) Relative K^+ flux mediated by the K^+ channel. (**E, K, Q**) Relative K^+ flux mediated by the H^+/K^+ symporter. (**F, L, R**) Relative K^+ flux mediated by the H^+/K^+ antiporter. The fluxes are shown relative to the maximal H^+ efflux that can be generated by the H^+ ATPase ($J_{Hmax} = I_{Hmax}/e_0$). Data were calculated for the case n_s = 1, n_a = 1, $V_{0,pump}$ = -150 mV. The magenta lines show the values in the absence of active H^+/K^+ antiporters (g_{HKa} = 0), whereas the cyan lines indicate the values in the absence of active H^+/K^+ symporters (g_{HKs} = 0).
