# Supplementary material for: Homeostats: The hidden rulers of ion homeostasis in plants
Source: Quant Plant Biol. 2024 Sep 3;5:e8. doi: 10.1017/qpb.2024.8 (PMC11706688; doi:10.1017/qpb.2024.8)
Supplement: Peer review history

# Editor report: Recommendation: Homeostats: The hidden rulers of ion homeostasis in plants — R0/PR2

**DOI:** 10.1017/qpb.2024.8.pr2

**Affiliations:** Biology, Utrecht University, Netherlands

No accompanying comment.

# Editor report: Decision: Homeostats: The hidden rulers of ion homeostasis in plants — R0/PR3

**DOI:** 10.1017/qpb.2024.8.pr3

**Affiliations:** RDP, ENS de Lyon, France

No accompanying comment.

# Editor report: Recommendation: Homeostats: The hidden rulers of ion homeostasis in plants — R1/PR5

**DOI:** 10.1017/qpb.2024.8.pr5

**Affiliations:** Biology, Utrecht University, Netherlands

No accompanying comment.

# Editor report: Decision: Homeostats: The hidden rulers of ion homeostasis in plants — R1/PR6

**DOI:** 10.1017/qpb.2024.8.pr6

**Affiliations:** RDP, ENS de Lyon, France

No accompanying comment.
